# Large language models reduce public knowledge sharing on online Q&A platforms

**DOI:** 10.1093/pnasnexus/pgae400

**Published:** 2024-09-11

**Authors:** R Maria del Rio-Chanona, Nadzeya Laurentsyeva, Johannes Wachs

**Affiliations:** Department of Computer Science, University College London, London WC1E 6EA, United Kingdom; Bennett Institute for Public Policy, University of Cambridge, Cambridge CB3 9DT, United Kingdom; Complexity Science Hub Vienna, Vienna 1080, Austria; Faculty of Economics, LMU Munich, Munich 80539, Germany; Sanofi Hungary, Budapest 1138, Hungary; Department of Network Science, Corvinus University of Budapest, Budapest 1093, Hungary; Institute of Economics, HUN-REN Centre for Economic and Regional Studies, Budapest 1097, Hungary; Complexity Science Hub Vienna, Vienna 1080, Austria

**Keywords:** AI, web, online public goods, ChatGPT

## Abstract

Large language models (LLMs) are a potential substitute for human-generated data and knowledge resources. This substitution, however, can present a significant problem for the training data needed to develop future models if it leads to a reduction of human-generated content. In this work, we document a reduction in activity on Stack Overflow coinciding with the release of ChatGPT, a popular LLM. To test whether this reduction in activity is specific to the introduction of this LLM, we use counterfactuals involving similar human-generated knowledge resources that should not be affected by the introduction of ChatGPT to such extent. Within 6 months of ChatGPT’s release, activity on Stack Overflow decreased by 25% relative to its Russian and Chinese counterparts, where access to ChatGPT is limited, and to similar forums for mathematics, where ChatGPT is less capable. We interpret this estimate as a lower bound of the true impact of ChatGPT on Stack Overflow. The decline is larger for posts related to the most widely used programming languages. We find no significant change in post quality, measured by peer feedback, and observe similar decreases in content creation by more and less experienced users alike. Thus, LLMs are not only displacing duplicate, low-quality, or beginner-level content. Our findings suggest that the rapid adoption of LLMs reduces the production of public data needed to train them, with significant consequences.

**Significance Statement**

This study examines the impact of ChatGPT, a large language model, on online communities that contribute to public knowledge shared on the Internet. We found that ChatGPT has led to a 25% drop in activity on Stack Overflow, a key reference website where programmers share knowledge and solve problems. This substitution threatens the future of the open web, as interactions with AI models are not added to the shared pool of online knowledge. Moreover, this phenomenon could weaken the quality of training data for future models, as machine-generated content likely cannot fully replace human creativity and insight. This shift could have significant consequences for both the public Internet and the future of AI.

## Introduction

Over the last 30 years, humans have constructed a vast and open library of information on the web. Using powerful search engines, anyone with an internet connection can access valuable information from online knowledge repositories like Wikipedia, Stack Overflow, and Reddit. New content and discussions posted online are quickly integrated into this ever-growing ecosystem, becoming digital public goods used by people all around the world to learn new technologies and solve their problems (1–4).

These public goods are essential for training AI systems, in particular, large language models (LLMs) (5). For example, the LLM in ChatGPT (6) is trained to recognize patterns, facts, and information from vast repositories of online public text by predicting the next words in sequences. It answers users’ questions by generating responses that not only integrate and contextualize this information but also infer underlying meanings and connections. The remarkable effectiveness of ChatGPT is reflected in its quick adoption (7) and application across diverse fields, including auditing (8), astronomy (9), medicine (10), and chemistry (11). Randomized control trials show that using LLMs significantly boosts productivity and quality in computer programming, professional writing, customer support tasks, consulting, and writing job applications (12–16). Indeed, the widely reported successes of LLMs, like ChatGPT, suggest that we will observe a significant change in how people search for, create and share information online.

Ironically, if LLMs like ChatGPT, substitute for traditional methods of searching and interrogating the web, they could displace the very human behavior that generated their original training data. As people begin to use ChatGPT or similar LLMs instead of online knowledge repositories to find information, traffic and contributions to these repositories will likely decrease, diminishing the quantity and quality of these digital public goods. Previous work refers to this sort of displacement as the “paradox of re-use”: for example, the information on platforms like Wikipedia powers Google search (via information boxes and summaries) while reducing the need to visit Wikipedia (17, 18). While such a shift could have significant social and economic implications, we have little evidence on whether people are indeed reducing their consumption and creation of valuable digital public goods as LLMs’ popularity grows.

The aim of this article is to evaluate the impact of LLMs on the generation of open data on popular question-and-answer (Q&A) platforms. We focus on the effects of the most widely adopted LLM as of now—ChatGPT. Because ChatGPT performs relatively well on software programming tasks (15), we study Stack Overflow, the largest online Q&A platform for software development and programming. Preliminary studies have shown that ChatGPT’s quality is competitive with answers from Stack Overflow in specific fields (19, 20).

We present three results. First, we examine whether the release of ChatGPT has decreased the volume of posts, i.e. questions and answers, published on the platform. We estimate the causal effect of ChatGPT’s release on Stack Overflow activity using a difference-in-differences model. We compare the weekly posting activity on Stack Overflow against that of four comparable Q&A platforms. These counterfactual platforms are less likely to be affected by ChatGPT either because their users experience difficulties with accessing ChatGPT or because ChatGPT performs poorly in questions discussed on those platforms.

We find that posting activity on Stack Overflow decreased by about 25% relative to the counterfactual platforms 6 months after the release of ChatGPT. We estimate the average effect across the 6 months to be 15%, reflecting a lagged kick-in and gradual adoption of ChatGPT. We interpret the 25% figure as a lower bound of the total impact of ChatGPT on Stack Overflow, as LLMs likely had some impact on even the counterfactual platforms. Additional evidence from the 2023 Stack Overflow Developer Survey supports the hypothesis that ChatGPT users are less likely to post on Stack Overflow and to visit the platform regularly.

Second, we investigate whether ChatGPT is simply displacing lower-quality posts on Stack Overflow. To do so, we use data on up- and downvotes, simple forms of social feedback provided by other users to rate posts. We observe little change in the votes posts received on Stack Overflow since the release of ChatGPT. In addition, we find significant declines in posting by users of all experience levels, from novice to expert. These results suggest that ChatGPT is displacing various Stack Overflow posts, including high-quality content.

Third, we study the heterogeneity of ChatGPT’s impact across different programming languages discussed on Stack Overflow. We test for these heterogeneities using an event study design. We observe that posting activity in some languages, like Python and Javascript, has decreased significantly more than the platform’s average. Using data on programming language popularity on GitHub, we find that the most widely used languages (and, hence, languages with richer data for training ChatGPT) tend to have larger relative declines in posting activity.

Our analysis points to several significant implications for the sustainability of the current AI ecosystem. The first is that the decreased production of open data will limit the training of future models (21). LLM-generated content itself is likely an ineffective substitute for training data generated by humans for the purpose of training new models (22–24). One analogy is that training an LLM on LLM-generated content is like making a photocopy of a photocopy, providing successively less satisfying results (25). While human feedback to LLMs may facilitate continued learning, data generated by interactions with privately owned LLMs belong to the owners of these LLMs.

This leads to the second issue: the initial advantage of the first mover, in this case OpenAI with its ChatGPT, compounds if the LLM effectively learns from interactions with users while crowding out the generation of new open data that competitors could use to improve their models. While it is well-known that increasing returns to users and data in the digital sector can lead to winner-take-all dynamics and technological lock-in (26, 27), the transformation of the online commons into a private database presents a novel risk to consumer welfare. More broadly, a shift from open data to a more closed web will likely have significant second-order impacts on the ever-growing digital economy (28) and how we access, share, and evaluate information. These potential consequences have been overlooked in previous risk taxonomies of LLMs (29).

The rest of the article is organized as follows. We introduce our empirical set-up, including the data and models used in our analysis, in Data and methods section. Results section presents our results. In Discussion section, we discuss their implications. We argue that our findings of a significant decline in activity on Stack Overflow following the release of ChatGPT have important implications for the training of future models, competition in the AI sector, the provision of digital public goods, and how humans seek and share information.

## Data and methods

### Stack exchange and Segmentfault data

To measure the effect ChatGPT can have on digital public goods, we compare the change in Stack Overflow’s activity with the activity on a set of similar platforms. These platforms are similar to Stack Overflow in that they are technical Q&A platforms but are less prone to substitution by ChatGPT given their focus or target group. Specifically, we study the Stack Exchange platforms: Mathematics and Math Overflow and the Russian-language version of Stack Overflow. We also examine a Chinese-language Q&A platform on computer programming called Segmentfault.

Mathematics and Math Overflow focus on university- and research-level mathematics questions, respectively. We consider these sites to be less susceptible to replacement by ChatGPT given that, during our study’s period of observation, the free-tier version of ChatGPT performed poorly (0–20th percentile) on advanced high-school mathematics exams (6) and was therefore unlikely to serve as a suitable alternative to these platforms.

The Russian Stack Overflow and the Chinese Segmentfault have similar scope as Stack Overflow, but target users located in Russia and China, respectively. We consider these platforms to be less affected by ChatGPT given that ChatGPT is officially unavailable in the Russian Federation, Belarus, Russian-occupied Ukrainian territory, and the People’s Republic of China. Although people in these places can and do access ChatGPT via VPNs, such barriers still represent a hurdle to widespread fast adoption (30).

We extract all posts (questions and answers) on Stack Overflow, Mathematics, Math Overflow, and Russian Stack Overflow from their launch to early June 2023 using https://archive.org/details/stackexchange. We scraped the data from Segmentfault directly. Our initial dataset comprises 58 million posts on Stack Overflow, over 900 thousand posts for the Russian-language version of Stack Overflow, 3.5 million posts on Mathematics Stack Exchange, 300 thousand posts for Math Overflow, and about 300 thousand for Segmentfault. We focus our analysis on data from January 2022 to the end of May 2023, noting that our findings are robust to alternative time windows.

For each post in the Stack Exchange sites, we additionally extract the post’s type (question or answer), the number of votes (up—positive feedback, or down—negative feedback) the post received, and the tags assigned to the post, where tags are predefined labels that summarize the content of the post, for instance, an associated programming language. In addition, we also extract the experience of the post’s author (i.e. number of previous posts). Using this information, we classify posts into those from “New”, “Inexperienced”, “Experienced”, and “Expert” users depending on whether the author had 0, 1–10, 11–100, or more than 100 posts, respectively at the time the post was published.^a^ For more details on the data from Q&A platforms we used, we refer the reader to section.

Finally, we also investigated data from the 2023 Stack Overflow Developer Survey, conducted in mid-May 2023. It includes 89,184 responses from software developers living in 185 countries. We focus on user responses to the prompt “Which AI-powered tools did you use regularly over the past year?”, for which ChatGPT was an option to tick. We use this information to provide further suggestive evidence for the relationship between the adoption of ChatGPT and Stack Overflow activity at the individual programmer’s level while controlling for a rich set of characteristics, such as professional status, education, experience, and preferred programming language.

### Models

#### Difference-in-differences

We estimate the effect of ChatGPT for posting activity on Stack Overflow using a difference-in-differences method with four counterfactual platforms. We aggregate posting data at platform- and week-level and fit a regression model using ordinary least squares (OLS):^b^


(1) $$\mathrm{Log}({\mathrm{Posts}}_{\;p,t})=\alpha_{\;p}+\lambda_{t}+\beta \times {\mathrm{Treated}}_{\;p,t}+\epsilon_{\;p,t},$$

where ${\mathrm{Posts}}_{\;p,t}$ is the number of posts on platform *p* in a week *t*, which we log-transform. $\alpha_{\;p}$ are platform fixed effects, $\lambda_{t}$ are time (week) fixed effects, and $\epsilon_{\;p,t}$ is the error term.

The coefficient of interest is *β*, which captures the estimated effect of ChatGPT on posting activity on Stack Overflow relative to the less affected platforms: Treated equals one for weeks after the release of ChatGPT (starting with the week of 2022 November 27) when the platform *p* is Stack Overflow and zero otherwise. We report standard errors clustered at the monthly level to account for month-specific shocks common to all platforms. We note that *β* defines an estimate of the effect of ChatGPT on Stack Overflow relative to the counterfactuals averaged across the entire 6-month post-treatment period of our data. We focus our difference-in-differences estimations on the period between January 2022 and May 2023, covering 48 weeks before the release of ChatGPT and 25 weeks after it.^c^ However, to show that our results are not specific to the selected time window, we also repeat the estimations using a wider time period starting from January 2019.

**Fig. 1.**
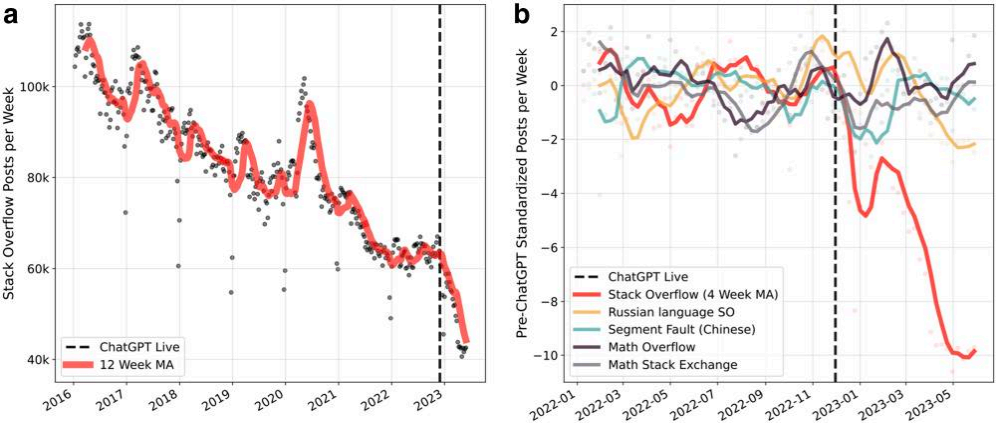
a) Time series of weekly posts to Stack Overflow since early 2016. The number of weekly posts decreases at a rate of about 7,000 posts each year from 2016 to 2022. In the 6 months after the release of ChatGPT, the weekly posting rate decreases by around 20,000 posts. b) Comparing posts to Stack Overflow, its Russian- and Chinese-language counterparts, and mathematics Q&A platforms since early 2022. Post counts are standardized by the average and standard deviation of post counts within each platform prior to the release of ChatGPT. Posting activity on Stack Overflow falls significantly more relative to activity on other platforms.

The validity of the difference-in-differences approach relies on the assumption of parallel trends. While Fig. 1b, illustrates that posting activity on Stack Overflow and the counterfactual platforms had developed in a similar way prior to the ChatGPT shock, we conduct several formal checks. First, we add platform-specific time trends that represent an interaction between a linear time trend and the average change in the number of posts on a platform between 2018 and pre-GPT. This allows us to check if the results are robust to the inclusion of differential time trends (32). Second, we estimate a generalized difference-in-differences model. Specifically, we employ a similar specification, but instead of $\beta \times {\mathrm{Treated}}_{\;p,t}$, we use $\sum_{t}\beta_{t}\times I(\mathrm{week}=t)\times I(\mathrm{platform}\;=\mathrm{StackOverflow})$. We standardize the effects to 0 in the week before the public release of ChatGPT by dropping the indicator for that week from the regression. This model allows us to examine possible pretrends in our data. By estimating separate coefficients for the weeks *before* the release, we can check if posts on Stack Overflow had evolved similarly to the activity on counterfactual platforms prior to the release of ChatGPT. This specification also allows us to investigate the dynamics of the ChatGPT effect over time. Separate coefficients for 25 weeks *following* the release of ChatGPT show how the effects of ChatGPT realized over time as more users adopted the technology.

The advantage of the difference-in-differences method compared to a simple event study with Stack Overflow data only is that we estimate ChatGPT effects net of possible weekly shocks that are common across the technical Q&A platforms. For the interpretation of the coefficient, we note that we estimate *relative* change in posting activity on Stack Overflow compared to activity on other platforms before vs. after the release of ChatGPT. To the extent that ChatGPT also affected activity on the counterfactual platforms, our estimates will be downward biased in the magnitude of the effect.

To investigate whether the decrease in posting was driven mainly by a decrease in the number of posts authored by new or inexperienced users, we run the same regression as in Eq. 1 separately for weekly posts made by users with different levels of prior experience. We assign each post the number of previous posts the user had made and differentiate between four groups of posts: posts by “new” users who have not posted before, posts by “inexperienced” users who posted between 1 and 10 times, posts by “experienced” users with between 11 and 100 prior posts, and posts by “expert” users who posted more than 100 times previously.

#### Event study

When analyzing the effect of ChatGPT on activity across programming languages, we can no longer compare data from Stack Overflow with the counterfactual platforms. This is because the tags annotating posts are different between Stack Exchange platforms. Therefore, we study ChatGPT’s heterogeneous effects using an event-study specification. For each programming language *i* (identified by a tag), we model the standardized number of posts in a week *t* on Stack Overflow by fitting a simple linear time trend with seasonal effects:


(2) $${\bar{\mathrm{Posts}}}_{i,t}=\beta_{0}+\beta_{1}t+\beta_{2}\mathrm{ChatGPT}+\beta_{3}(t\times \mathrm{ChatGPT})+\eta +\epsilon_{i,t},$$

where $\bar{{\mathrm{Posts}}_{i,t}}$ stands for the standardized number of posts associated with a programming language *i* in a week *t*. We standardize the dependent variable in order to be better able to compare effects across programming languages with different numbers of posts.^d^$\beta_{1}(t)$ captures the linear time trend and *η* are seasonal (month of year) fixed effects. ChatGPT equals one if the week *t* is after the release of ChatGPT and zero otherwise. Coefficient $\beta_{2}$ captures the change in the intercept, while coefficient $\beta_{3}$ reflects the change in the slope of the time trend following the release of ChatGPT. We report HAC standard errors.

#### Additional regression analysis with the Stack Overflow 2023 survey

We run an additional model using Stack Overflow survey data to corroborate our findings. We compute the association between self-reported individual activity on Stack Overflow and the adoption of ChatGPT by estimating the following logistic regression:


(3) $$\begin{array}{rl} \log(\frac{{\mathrm{Activity}}_{d,i}}{\left(1-{\mathrm{Activity}}_{d,i}\right)}) & =\beta_{0}+\beta_{1}({\mathrm{Use}\;\mathrm{ChatGPT}}_{d})\\ & \;+\beta_{2}(X_{d})+{\mathrm{Age}}_{d}+C_{d}+I_{d}+{\mathrm{Lang}}_{i}+{\mathrm{Type}}_{d}+\epsilon_{d,i}, \end{array}$$

where *d* stands for the developer and *i* denotes a programming language used by the developer. ${\mathrm{Activity}}_{d,i}$ corresponds to the probability of being a frequent Stack Overflow visitor/contributor. $X_{d}$ comprises a set of controls at the developer’s level: a dummy of whether the developer is a professional software engineer, education level, employment status, working mode (remote, hybrid, or in-person), and years of coding. We also add age (Age), country (*C*), industry (*I*), programming language (Lang), and developer type (Type) (e.g. researcher, front-end, back-end, full-stack, QA, etc.) fixed effects. Because most developers report using more than one programming language, we expand the dataset to the developer × language level. We apply weights (1/number of languages) to avoid double counts.^e^ We cluster standard errors at the programming language level to allow for common shocks. While the cross-sectional nature of the survey data does not allow us to interpret the results as causal, we try to reduce the endogeneity by controlling for a rich set of the above individual characteristics that are likely to influence both the adoption of ChatGPT and Stack Overflow contributions. In this way, we are comparing how the contributions to Stack Overflow vary between ChatGPT adopters and nonadopters, who are otherwise very similar to each other.

## Results

### Decrease in posting activity

Figure 1a shows the evolution of activity on Stack Overflow from January 2016 to June 2023. Up to 2022 there was a gradual decrease in activity from roughly 110,000 to 60,000 posts per week, that is roughly 7,000k posts less per week each year. However, after the release of ChatGPT (2022 November 30) posting activity decreased sharply, with the weekly average falling from around 60,000 posts to 40,000 within 6 months. Compared to the pre-ChatGPT trend, this decrease represents more than 5 years worth of deceleration in just half a year.

The decrease in activity on Stack Overflow is larger than for similar platforms for which we expect ChatGPT to be a less viable substitute. Figure 1b shows the standardized posting activity on Stack Overflow, the Russian- and Chinese-language counterparts of Stack Overflow, and two mathematics Q&A platforms. We standardize posting activity by the average and standard deviation of post counts within each platform prior to the release of ChatGPT.

Figure 1b highlights that Stack Overflow activity deviates markedly from activity on the other platforms after the release of ChatGPT. The plot visualizes the standardized posting activity within each platform since early 2022. Smoothed weekly activity varies between plus and minus two standard deviations for all platforms for most of 2022. Events, such as the Chinese New Year and other holidays and the start of the Russian invasion of Ukraine, are visible. Following the release of ChatGPT, we observe a significant and persistent decline in activity on Stack Overflow.

Our difference-in-differences model reveals that Stack Overflow activity significantly declined after the release of ChatGPT, and that this effect became more pronounced over time. Table 1 reports our estimates, the first column indicates that ChatGPT decreased posting activity on Stack Overflow by 15% ($1-e^{-0.163}$). Note that this is a measure of the average effect across the 6 months of post-ChatGPT data we consider. If ChatGPT adoption is gradual, we expect that the effect observed at the end of the data will be larger than at the beginning.

**Table 1.** Results of a difference-in-differences model, estimating the change in activity observed weekly on stack overflow following the release of ChatGPT, relative to activity on four other platforms less likely to have been impacted.

	(1)	(2)	(3)
	Number of posts	Number of questions	Weekday posts
Variables			
Stack Overflow × Post-GPT	$-0.163^{*}$	−0.105+	$-0.151^{*}$
	(0.0584)	(0.0597)	(0.0613)
Observations	370	370	370
$R^{2}$ -within	0.0458	0.0189	0.0294

All regressions comprise platform fixed effects and week fixed effects. The standard error of the estimate clustered on month is reported in parentheses. $R^{2}$ (within) is derived after differencing out week and platform fixed effects. Significance codes: ***: P<0.001, ^**^: P<0.01, ^*^: P<0.05, +: P<0.1.

**Fig. 2.**
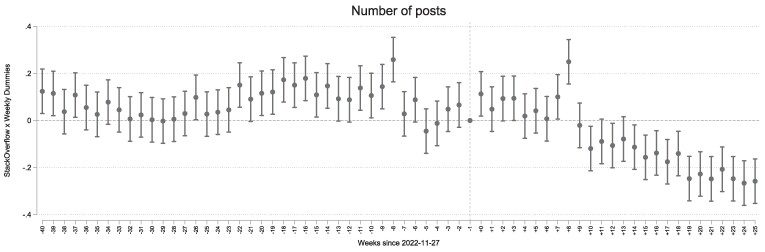
Difference-in-differences analysis for posting activities. The dashed line marks the week of 2022 November 27—the release week of ChatGPT. Eight weeks after its introduction, we observe a steady decline in the activity of Stack Overflow. The plotted coefficients correspond to the interaction between a weekly dummy and posting on Stack Overflow. We normalize the effects to 0 in the week before the public release of ChatGPT by dropping the indicator for that week from the regression. The reported CIs are at 95%. The regression comprises platform fixed effects and week fixed effects.

Indeed, our second specification observes exactly this trend. We visualize the weekly estimates of the relative change in the Stack Overflow activity in Fig. 2. This figure shows the impact of ChatGPT is increasing over time and is greater in magnitude than the average post-ChatGPT effect estimated in Table 1 by the end of our study period. By the end of April 2023, coinciding with a peak in traffic to ChatGPT, ^f^ the estimated decrease in activity stabilizes at around 25%.

We also tested for heterogeneity in subsets of the data, considering only questions (rather than counting both questions and answers) and posts on weekdays. In both subsets, our estimates did not deviate significantly from the main result: we estimate a 10% relative decrease in questions and 14% relative decrease in posts on weekdays (see the second and third column of Table 1). Our results are robust to using alternative transformations of the outcome (Tables S1 and S2), adding platform-specific trends (Table S3), and extending the time window of the analysis (Table S4).

The decrease in Stack Overflow activity is consistent with the individual-level evidence from the 2023 Stack Overflow Developer Survey. In particular, we estimate the relationship between self-reported ChatGPT usage and Stack Overflow visit and contribution frequency using specification Eq. 3. We consider several binary variables as the outcomes: *Contribute to Stack Overflow ever (weekly or more)* is equal to one if a developer has contributed to Stack Overflow at least once (weekly or more often) and zero otherwise, *Visit Stack Overflow daily* is equal to one if a developer reports visiting the platform once or more times per day. As participants were recruited through Stack Overflow and related platforms, they represent a selected group of engaged Stack Overflow users, and, therefore, their levels of activity are high: about 75% of all respondents have contributed to the platform at least once, and 42% visit it daily. We report the results in Table S7. The coefficients represent changes in log odds of the outcomes, and we also compute the average marginal effects of ChatGPT adoption on the likelihood of frequent contributions/visits.

We find that ChatGPT adopters are less likely to contribute to Stack Overflow and to visit the platform frequently compared to nonadopters of the same age, experience, education, employment status, working mode, industry, and programming language used. Moreover, even when we limit the sample to the most active respondents (i.e. those who have contributed at least once to Stack Overflow), we can still detect statistically significant differences in the probability of both contributing weekly and visiting daily between otherwise similar ChatGPT adopters and nonadopters. The magnitude of the effect is not very high. For instance, the average marginal effect of ChatGPT on the likelihood of contributing to Stack Overflow weekly or more is about 0.8 percentage points (or 2.7% lower probability). However, these results are likely to be downward biased because of the selection into survey participation: those who use Stack Overflow less frequently (including those who have reduced their activity because of ChatGPT) were less likely to respond.

#### Post and user heterogeneities

A decrease in overall activity on Stack Overflow is not an issue if it is rather the less interesting questions that are outsourced to ChatGPT. We use a post’s score (i.e. difference between upvotes and downvotes) observed 5 weeks after its creation as a proxy of its value—good (bad) posts have a positive (negative) score, while neutral questions have a score of zero.

**Fig. 3.**
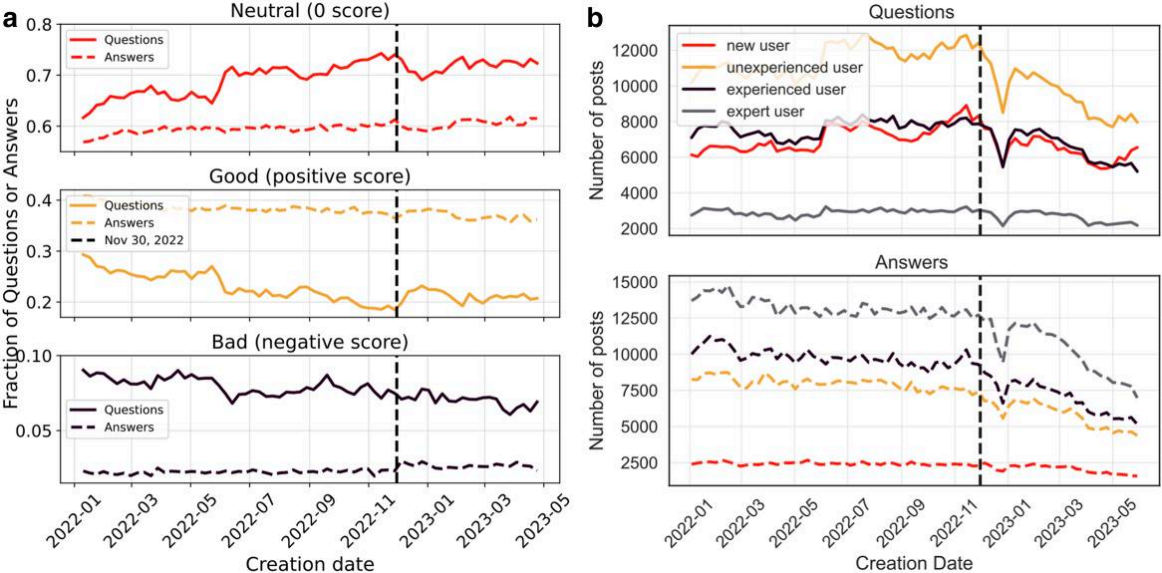
a) The weekly time series of the fraction of neutral, good, and bad questions and answers. Good (bad) post are those with a positive (negative) score after 5 weeks of its creation, while neutral questions have a zero score. The horizontal axis indicates the week of the post and the dashed line the week of the release of ChatGPT. We observe no major change in trends in bad posts since the release of ChatGPT. b) Weekly counts of questions and answers, respectively, by users, binned by experience level at the time of posting. We observe larger decreases in posting by users with previous experience post-ChatGPT.

If ChatGPT is displacing bad questions, we would expect that after its release there would be a downward trend in the share of bad questions. However, as Fig. 3a shows, while there was a slight uptick in the fraction of good questions, these were mostly replacing neutral questions and the trend of bad questions was flat. The short-lived increase in the fraction of good questions may be a result of ChatGPT inducing interest in novel topics, such as large language models, which usually results in good questions (see our Discussion section below on the increase in interest in CUDA). With respect to answers, there was no change in the trends of good, bad, and neutral answers. In general, there is a remarkable stability in the proportions post-ChatGPT. We confirm these results by estimating a difference-in-differences specification where the outcome is the number of up(down) votes that posts published in a given week receive over the first 5 weeks, normalized to the total number of posts from this week (Table S5 and Fig. S1). Unlike our previous results on posts, we do not detect any effect of ChatGPT.

Votes do not capture all aspects of quality or more generally the ways in which ChatGPT may have influenced content on Stack Overflow. For example, users with different levels of experience contribute different kinds of content to the platform. New users tend to ask more basic questions, which ChatGPT may answer better. In contrast, experienced users may ask more sophisticated questions beyond the abilities of ChatGPT. A heterogeneous effect of ChatGPT on participation on Stack Overflow by users stratified by experience would have significant implications for content.

Table 2 reports changes in activity estimated in a difference-in-differences specification, decomposed by prior user experience at the time of posting. Our estimates show that, while posts made by first-time users on Stack Overflow decreased only slightly relative to the control platforms, inexperienced, experienced, and expert users made significantly fewer posts on average after the release of ChatGPT.^g^ The point estimates (a reduction of about 21% relative to the counterfactual platforms) for both inexperienced and experienced users are almost identical, suggesting no significant difference in the decrease in activity. Table S8 estimates separate effects of ChatGPT on questions and answers. Interestingly, while inexperienced users reduce the number of their questions and answers to a similar extent, the effects for experienced and expert users are more pronounced for posting answers. We could link the latter result to lower incentives to contribute to Stack Overflow: as fewer developers are using the platform, the visibility “premium” that could be earned by answering questions becomes lower.

**Table 2.** Results of a difference-in-differences model, estimating the change in activity observed weekly on stack overflow following the release of ChatGPT by user group, relative to activity on three other platforms (we exclude segment fault as we do not have access to user experience data) less likely to have been impacted.

	(1)	(2)	(3)	(4)
	Number of posts	Number of posts	Number of posts	Number of posts
VARIABLES	NewUser	InexperiencedUser	ExperiencedUser	ExpertUser
Stack Overflow × Post-GPT	$-0.0833^{*}$	$-0.245^{***}$	$-0.254^{***}$	$-0.168^{***}$
	(0.0376)	(0.0529)	(0.0424)	(0.0292)
Observations	296	296	296	296
$R^{2}$ -within	0.0259	0.198	0.235	0.104

Posts by new users are posts by users with no previous posts at the time of posting. Inexperienced users have posted 1–10 times before, experienced users 11–100, and experts more than 100 times. All regressions comprise platform fixed effects and week fixed effects. The standard error of the estimate clustered on month is reported in parentheses. $R^{2}$ (within) is derived after differencing out week and platform fixed effects. Significance codes: ***: P<0.001, ^**^: P<0.01, ^*^: P<0.05, +: P<0.1.

Overall, our analysis shows little evidence that ChatGPT tends to replace low-quality posts and no evidence that it replaced posts by inexperienced users relative to experts and experienced users.

### Heterogeneities across programming languages

**Fig. 4.**
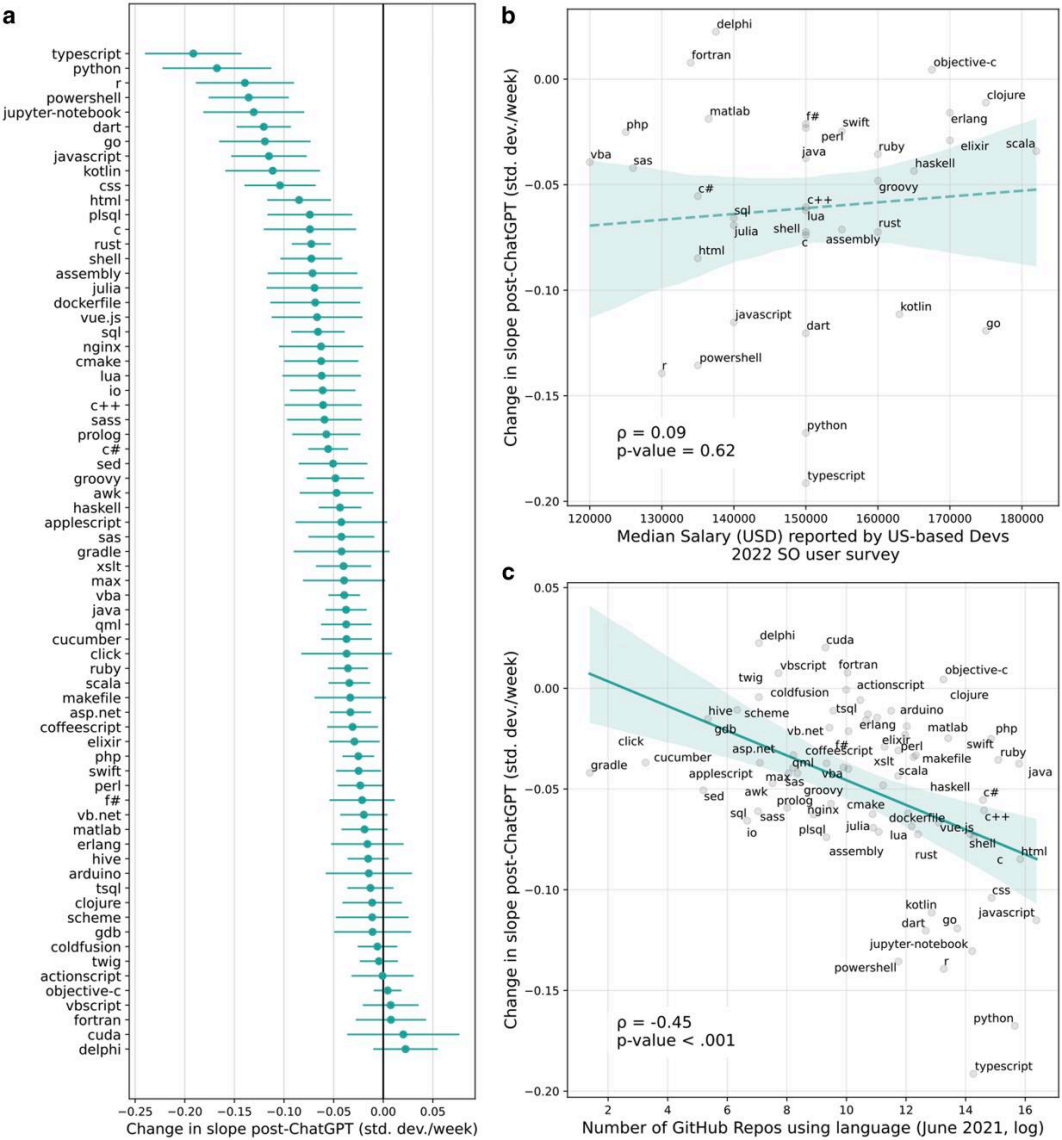
a) The event study estimates of the effect of ChatGPT’s release on activity on a selection of tags on Stack Overflow. We report HAC-corrected 95% CIs. b) The relationship between estimated effects and salary data from the Stack Overflow Developer Survey. We find no significant relationship. c) The relationship between the number of GitHub repositories using a tag and the estimated effect of ChatGPT on that tag. In both b) and c), we plot a linear fit with bootstrapped 95% CIs. The dashed line in b) indicates that the correlation is not significant.

Next, we investigated differences in the impact of ChatGPT on posts about different programming languages, finding significant heterogeneities. In Facet A of Fig. 4, we plot the estimated effects (slope changes in the linear time trend after the introduction of ChatGPT) for those 69 tags that we connected to a programming language on GitHub. We estimate a negative effect of ChatGPT for most tags, but the estimates range between a 0.25 standard deviation decrease in slope (i.e. change per week following the ChatGPT release) to a 0.03 standard deviation *increase*. We observe that some of the widely used languages like Python and Javascript are the most impacted by ChatGPT. Interestingly, the model estimates that posts about CUDA have increased (though not significantly) after ChatGPT was released. CUDA is an application programming interface created by Nvidia, a graphics card manufacturer, that facilitates the use of graphics cards for computational tasks, in particular for machine learning and AI. This exception again demonstrates the impact of ChatGPT on the world of computer programming: people are increasingly interested in software relating to AI.

Given that previous research suggests that high-wage jobs are more exposed to ChatGPT (33), we test whether the impact of ChatGPT is more predominant among better-paid languages. We source salary data from the 2022 Stack Overflow Developer Survey, focusing on US-based developers and calculating medians of reported salaries. In Fig. 4b, we compare the estimated impact of ChatGPT on different languages against the salary data of developers using those languages. We observe no clear relationship between the estimated labor market value of a specific language and changes in posting behavior in that language post-ChatGPT.

To better understand the relationship between the size of the user base of a programming language and how it is impacted by ChatGPT, we compare our estimates with data from GitHub, the largest online platform for collaborative software development. Among other sources, ChatGPT was trained on data from GitHub. Because training data were collected up to September 2021, we use data on language use on GitHub up to June 2021. In Facet C of Fig. 4, we visualize the relationship between the number of GitHub repositories (coding projects) in a specific language and the estimated impact of ChatGPT on that language. We observe that languages with more GitHub repositories tend to be more significantly impacted by the release of ChatGPT in terms of associated activity on Stack Overflow (Pearson’s ρ=−0.45,P<0.001). This result is confirmed by estimating a difference-in-differences specification that compares the change in posting following the release of ChatGPT between more and less popular programming languages as measured by the number of GitHub commits attributed to a given language as of 2021 (Table S6).

### Subsequent dynamics

**Fig. 5.**
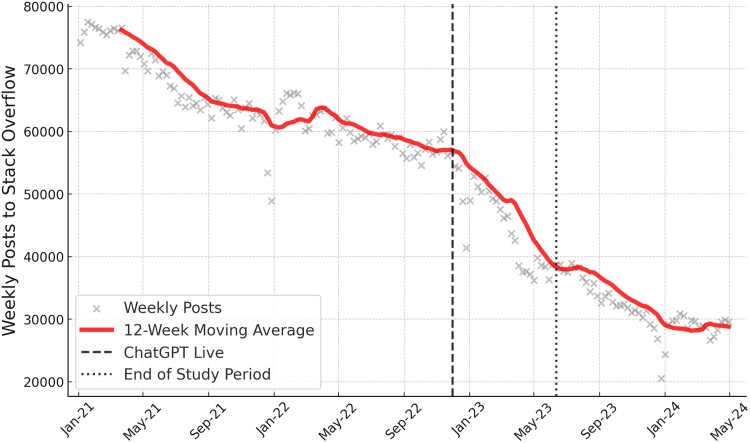
An extended timeseries of the weekly posts to Stack Overflow. We highlight the release of ChatGPT and the conclusion of the data we use in the statistical analyses, respectively. After May 2023, the decline in posting activity continues, albeit at a slower rate.

Using the Stack Exchange Data Explorer, we extended the timeseries of weekly posts to Stack Overflow to Spring 2024. We visualize this data in Fig. 5. We observe a continued, if slower, decrease in weekly posting activity after the end of our statistical analyses. In raw terms, the number of weekly posts to Stack Overflow has fallen from 60,000 to 30,000 from May 2022 to May 2024, with much of that change happening in the 6 months following the release of ChatGPT. Again, this suggests that the 25% estimate of the effect of ChatGPT on Stack Overflow should be interpreted as a lower bound effect, which is likely still growing.

An extension of the difference-in-differences analysis would not yield reliable estimates of the relative impact of LLMs of Stack Overflow for several reasons. First, the subsequent proliferation of ChatGPT or-better quality LLMs, including open source models and models available in Russia and China mean that the reference timeseries are no longer valid counterfactuals. Moreover, advances in LLM capabilities have significantly improved their performance in mathematical tasks. Thus, we do not extend our difference-in-differences analyses.

## Discussion

The rate at which people have adopted ChatGPT is one of the fastest in the history of technology (7). It is essential that we better understand what activities this new technology displaces and what second-order effects this substitution may have (34, 35). This article shows that after the introduction of ChatGPT there was a sharp decrease in human content creation on Stack Overflow. We compare the decrease in activity on Stack Overflow with other Stack Exchange platforms where current LLMs are less likely to be used. Using a difference-in-differences model, we estimates a 25% decline to posts on Stack Overflow relative to the counterfactual platforms within 6 months of ChatGPT’s release. We interpret this as a lower bound as ChatGPT is likely to have had small but growing impact on the counterfactual platforms as well. The Stack Overflow Developer Survey confirms that people using ChatGPT were less likely to post questions or answers on Stack Overflow.

We observe no large change in social feedback on posts, measured using votes, nor in the experience composition of posting users following ChatGPT’s release. These results suggest that average post quality has not changed, nor has ChatGPT replaced only the new and inexperienced users. Posting activity related to more popular programming languages decreased more on average than that for more niche languages. Given that LLMs performance depends on the quantity of training data, this finding suggests that users are more likely to substitute Stack Overflow with ChatGPT with respect to languages LLMs are more knowledgeable about. Consequently, the widespread adoption of LLMs will likely decrease the provision of digital public goods including open data previously generated by interactions on the web.

Two of our results offer some limited reasons for optimism. While posting activity on Stack Overflow decreased among inexperienced, experienced, and expert users relative to the control platforms, content created by new users remained relatively stable. New users are known to be essential to the long-run health of online communities (36). However, this optimism should be nuanced given that, if new users start behaving as inexperienced users did, then new users will also be more to likely reduce their activity in Stack Overflow. The second is that the impact of ChatGPT was less on more niche languages used by fewer people, suggesting that online conversations around such languages and the valuable information they generate will continue.

Recent work by Burtch et al. (37) studying the evolution of activity on Stack Overflow and Reddit found similar results to ours. Using a synthetic control method to adjust for seasonality, the authors report a roughly 20% decrease in posting activity on Stack Overflow within 15 weeks of the release of ChatGPT, and find similar heterogeneities among programming languages. These findings complement ours, which are derived from a more conservative analysis using counterfactual platforms. One difference in our findings is that their method finds a sharp decrease in posts by new users, while we observe fewer posts by more experienced users on Stack Overflow compared to the counterfactual platforms. It would be valuable for future work to resolve this ambiguity given the importance of new users to platform health discussed above.

Our results and data have some shortcomings that point to other open questions about the use and impact of LLMs. First, while we can present strong evidence that ChatGPT decreased the posting activity in Stack Overflow, we can only partially assess quality of posting activity using data on upvotes and downvotes. Users may be posting more challenging questions, ones that LLMs cannot (yet) address, to Stack Overflow. Future work should examine whether continued activity on Stack Overflow is more complex or sophisticated on average than posts from prior to ChatGPT release. Similarly, ChatGPT may have reduced the volume of duplicate questions about simple topics, though this is unlikely to impact our main results as duplicates are estimated to account for only 3% of posts (38), and we do not observe significant changes in voting outcomes.

A second limitation of our work is that we cannot observe the extent to which Russian- and Chinese-language users of the corresponding Q&A platforms are actually hindered from accessing ChatGPT; indeed recent work has shown a spike in VPN and Tor activity following the blocking of ChatGPT in Italy (30). While our results are robust to excluding the Chinese and the Russian counterfactuals, given the potential economic importance of ChatGPT and similar LLMs, it is essential that we better understand how such bans and blocks impact the accessibility of these tools (39, 40). Finally, we do not address the issue that ChatGPT may be used to generate Stack Overflow content. Stack Overflow policy effectively banned posts authored by ChatGPT within a week of its release. In any case, a significant amount of ChatGPT generated content on Stack Overflow would mean that our measures underestimate the magnitude of the ChatGPT effect.

Despite these shortcomings, our results have important implications for the future of digital public goods. Before the introduction of ChatGPT, more human-generated content was posted to Stack Overflow, forming a collective digital public good due to their nonrivalrous and nonexclusionary nature—anyone with internet access can view, absorb, and extend this information, without diminishing the value of the knowledge. Now, part of this information is rather fed into privately owned LLMs like ChatGPT. This represents a significant shift of knowledge from public to private domains.

This observed substitution effect also poses several issues for the future of AI. The first is that if language models crowd out open data creation, they will be limiting their own future training data and effectiveness. The second is that owners of the current leading models have exclusive access to user inputs and feedback, which, with a relatively smaller pool of open data, gives them a significant advantage against new competitors in training future models. Third, the decline of public resources on the web would reverse progress made by the web toward democratizing access to knowledge and information. Finally, the consolidation of humans searching for information around one or a few language models could narrow our explorations and focus our attention on mainstream topics. We briefly elaborate on these points, then conclude with a wider appeal for more research on the political economy of open data and AI, and how we can incentivize continued contributions to digital public goods.

###  

#### Training future models

Our findings suggest that the widespread adoption of ChatGPT may ironically make it difficult to train future models (41). Though researchers have already expressed concerns about running out of data for training AI models (21), our results show that the use of LLMs can slow down the creation of new (open) data. Given the growing evidence that data generated by LLMs are unlikely to effectively train new LLMs (22, 23), modelers face the real problem of running out of useful data. While research on using synthetic data and mixed data to train LLMs is still ongoing, current results show that use of synthetic training data can degrade performance (24) and may even amplify biases in models (42). Human input and guidance can mitigate these issues to some extent, but in general it is still unclear if synthetic data can power continued advances in LLM capabilities.

If ChatGPT truly is a “blurry JPEG” of the web (25), then in the long run, it cannot effectively replace its most important input: data derived from human activity. Indeed, OpenAI’s recent strategic partnerships with Stack Overflow and Reddit demonstrate the value of this kind of data for the continued training of LLMs.^h^ The proliferation of LLMs has already impacted other forms of data creation: many Amazon Mechanical Turk workers now generate content (i.e. respond to surveys, evaluate texts) using ChatGPT (43). And though watermarks may help humans and models identify data creators (44), the general problem of determining whether, for example, a text is written by a human or LLM is difficult at scale (45).

### Competition in the AI sector

A firm’s early advantage in technological innovation often leads to significant market share via various mechanisms of path dependence (46). There are increasing returns to using ChatGPT as more people use it, as it can learn from user feedback (26). Our results indicate that ChatGPT is simultaneously decreasing the amount of open training data that competitors could use to build competing models while it captures user data for itself, which may lead to technological lock-in (27). Unlike synthetic data, data on user interactions with LLMs can be used to significantly improve and tune their performance (47). We suggest that besides increasing returns to scale from network effects, the transformation of public data commons into private databases presents another mechanism by which the tech sector can become even more concentrated.

### Lost economic value

Digital public goods generate value in many ways besides feeding LLMs and other algorithms. For instance, Wikipedia is an important source of information worldwide, but in developing countries, readers are more often motivated by intrinsic learning goals and tend to read articles in greater detail (3). Unequal access to AI may also compound inequalities in growth and innovation between countries (40).

Digital public goods also provide direct value to the many websites that extract data from open data to complement their core services with extra information (4). For instance, there is substantial interdependence between sites like Wikipedia, Reddit, and Stack Overflow and the search engines that use them to enrich responses to user queries via infoboxes (17, 48), sometimes referred to as the “paradox of re-use” (18). In the case of search engines, putting links to knowledge sources within infoboxes has mitigated the issue to some degree (49), but LLMs like ChatGPT are substituting for search engines and are much less likely to link to sources. Their widespread adoption presents a significant threat to the overall sustainability of the web (50).

Creators of digital public goods may also lose out. Contributors to Stack Overflow or Open Source Software (OSS) often enjoy indirect benefits (51). For instance, while OSS itself provides significant value in the global economy (52), OSS contributions are valuable signals of a firm’s capabilities to investors (53). Individual contributions to Stack Overflow are used to signal ability on the labor market (54). Any general tendency of ChatGPT to crowd out contributions to digital public goods, may limit these valuable signals that reduce economic frictions. On the other hand, such signaling activity may serve as a powerful incentive to keep people contributing.

#### Narrowing of information seeking

The substitution effect we report likely has important second-order effects on how people search for information and their exposure to new ideas. LLMs likely favor well-established perspectives and due to their efficiency decrease the need for users to forage for information. These features of LLMs may reinforce a trend observed earlier in the context of the web. Specifically, internet search engines are thought to have pushed science toward consensus and narrower topics by improving efficiency of information search and improving the visibility of mainstream information (55). LLMs may also disincentivize the use of new or niche tools because they most amplify our productivity with those tools for which it has much training data. For instance, ChatGPT may not be able to help users of a new programming language that is has not seen many examples of. Given that LLMs are poised to change how we do research (56), present a strong competitor to search engines (57), and will likely influence our news consumption (58), we need to understand what LLM efficiency implies for our contact with diverse sources of information and incentives to try new things.

More generally, models like ChatGPT are going to generate political and economic winners and losers like many previous breakthrough technologies. While early evidence shows that these models enhance productivity especially among new and inexperienced workers (12, 14), there are other ways in which they may contribute to inequality between people and firms (59), for instance via potential negative side effects of automation (33, 60). Our results suggest that the economics of data creation and ownership will become more salient: as data become more valuable, there will be growing interest in how creators of data can capture some of that value (61). These multifaceted aspects of the impact of LLMs suggest that the political economy of data and AI will be especially important in the next years (58, 62, 63).

In this context, our work highlights the specific issue that valuable digital public goods may be under-produced as a result of the proliferation of AI. A natural follow-up question is how we can incentivize the creation of such goods. While unemployment shocks are known to increase the provision of digital public goods (64), it would be an unsatisfying solution to suggest that people put out of work by automation will fill this gap. In the case of platforms like Stack Overflow, active users are often motivated by social feedback and gamification (65), but the continual onboarding of new users is what keeps these platforms relevant in the long run (36). For the sake of a sustainable open web and an AI ecosystem that draws on its data, we should think about how to keep people exchanging information and knowledge online.

## Materials

###  

#### Stack Exchange platform sites

The raw dataset obtained from https://archive.org/details/stackexchange contains nearly all posting activity on the question and answer platforms hosted on the Stack Exchange network from its launch in 2008 to early June 2023. These include Stack Overflow, its Russian language version, and Math Overflow and Math Stack Exchange. Stack Overflow is the largest online Q&A platform for topics relating to computer programming and software development. It provides a community-curated discussion of issues programmers face (65). Questions have multiple answers, and users debate the relative merits of solutions and alternatives in comments. A track record on Stack Overflow has value on the labor market as a signal of an individual’s skills (54).

The data contain over 58 million posts, including both questions and answers. Posts are linked to their posting users, from which we infer poster previous activity and can identify posts made by new users. Questions are annotated with tags indicating the topic of the post including programming languages used. Users can give posts upvotes or downvotes, providing posting users with social feedback and reputation points. The Russian language version of Stack Overflow (over 900 thousand posts) and the mathematics-oriented platforms Math Stack Exchange (over 3.5 million posts) and Math Overflow (over 300 thousand posts) have identically structured data dumps hosted in the same location.

Registered users can upvotes and downvote posts made on Stack Exchange platforms. These votes provide a valuable signal of the value of posts (65, 66). They are the primary way users earn reputation points and status on Stack Exchange platforms. Votes also influence the ranking of posts in user feeds and search engine results, facilitating information filtering. Downvotes are used to moderate. The Stack Exchange data dump contains data on every vote cast, including the corresponding post, the date the vote was made, and whether it was an upvote or downvote.

#### Segmentfault

Segmentfault is a Chinese language platform with a Q&A platform for developers that has many similarities with the Stack Exchange sites. Users post questions on programming language topics and other users post answers. Questions are tagged by relevant languages and technologies, and there are similar gamification elements on the platform. We scraped data on all posts as of early June 2023, gathering over 300 thousand in total. We were careful to follow best practices when collecting this data, limiting strain on the host platform’s servers and retaining only anonymized data and metadata rather than content of posts (67).

#### Selection of tags

Stack Overflow posts are annotated by tags which describe the concepts and technologies used in the post. For example, many tags indicate programming languages, web frameworks, database technologies, or programming concepts like functions or algorithms. Stack Overflow reconciles tags referring to the same things via a centralized synonym dictionary. We selected the 1,000 most used tags up to early June 2023 and focused on those 69 which could be directly linked to language statistics reported by GitHub, described next.

#### GitHub data on programming language use

We use data from the June 2021 GHTorrent data dump (68) as a proxy measure for the amount of open data available for each programming language. The dataset reports which languages are used in each project or repository on GitHub. We simply count the number of repositories mentioning each language. We then link the languages with tags on Stack Overflow. As an alternative, we count the number of commits, elemental code contributions to repositories, to each repository, hence language. In the main article, we visualize the estimated effects of ChatGPT on specific tags that we can link to GitHub languages. We exclude some tags which refer to file formats or plain text, specifically: yaml, json, text, svg, markdown, and xml.

#### Stack Overflow Developer Survey

The 2023 Stack Overflow Developer Survey was conducted from 2023 May 8 to May 19 and captured responses from 89,184 software developers across 185 countries. Respondents were recruited primarily through channels owned by Stack Overflow, therefore users that are highly engaged on Stack Overflow were more likely to notice the prompts to take the survey over the duration of the collection promotion.^i^ This survey includes self-disclosed information about respondents professional status, academic qualifications, employment type, remote work status, and years of coding experience. Moreover the survey asked participants ’Which AI powered tools did you use regularly over the past year’ and included ChatGPT as an option to tick.

## Supplementary Material

pgae400_Supplementary_Data

## Data Availability

Data and code to reproduce our analyses are available on Zenodo: https://zenodo.org/records/12670482. The Stack Overflow data dump is available here: https://archive.org/details/stackexchange.

## Notes

a There is no standard classification of user experience based on the number of posts. We chose a log-binned classification since activity on Stack Overflow is heavy-tailed (31), and log base 10 is a commonly used base.

b For robustness, we test an OLS specification with standardized outcomes and a specification with the raw count of posts that we fit using the Poisson pseudo-maximum likelihood method.

c In this way, we do not include Covid-induced positive shock in 2020 and then the reversion to the trend in 2021.

d We standardize the number of posts within each tag by subtracting the mean and dividing by the standard deviation. Both statistics are calculated using data up to the release of ChatGPT.

e For example, if a developer reports using three languages, there will be three entries (one for each language) in our dataset for this developer, each with a weight of 1/3.

f
https://www.similarweb.com/blog/insights/ai-news/chatgpt-bard/

g Prior to the release of ChatGPT, new users contributed 9.5 thousand posts per week; inexperienced users—almost 20 thousand; experienced users—about 17.5 thousand, and expert users—16 thousand.

h See: https://www.theverge.com/2024/5/6/24150341/openai-stack-overflow-partner-api-coding-assistance and https://time.com/6979197/reddit-openai-partnership-chatgpt/

i Additional information about the survey and its methodology is available here: https://survey.stackoverflow.co/2023/

## Preprints

A preprint of this article is published at https://arxiv.org/abs/2307.07367.
