# Supplementary material for: Large language models reduce public knowledge sharing on online Q&A platforms
Source: PNAS Nexus. 2024 Sep 11;3(9):pgae400. doi: 10.1093/pnasnexus/pgae400 (PMC11421660; doi:10.1093/pnasnexus/pgae400)
Supplement: pgae400_Supplementary_Data [file pgae400_supplementary_data.pdf]

Large language models reduce public knowledge sharing on online Q&A platforms

R. Maria del Rio-Chanona [1,2,3], Nadzeya Laurentsyeva [4], and Johannes Wachs [5,6,3,*]

[1] University College London, London

[2] Bennett Institute for Public Policy, University of Cambridge

[3] Complexity Science Hub, Vienna

[4] Sanofi

[5] Corvinus University of Budapest

[6] Centre for Economic and Regional Studies, Hungary [*]

Supplementary Information

VARIABLES	(1) Number of posts	(2) Number of questions	(3) Weekday posts
Stack Overflow × Post-GPT	-0.873***	-0.757**	-0.768***
	(0.172)	(0.237)	(0.164)
Observations	370	370	370
R2-within	0.166	0.0842	0.131

Table S1: Results of a difference-in-differences model (with standardized instead of log outcomes), estimating the change in activity observed weekly on Stack Overflow following the release of ChatGPT, relative to activity on four other platforms less likely to have been impacted. The coefficients represent the change in the units of a standard deviation. All regressions comprise platform fixed effects and week fixed effects. The standard error of the estimate clustered on month is reported in parentheses. R-squared (within) is derived after differencing out week and platform fixed effects. Significance codes: ***: $p < 0.001$, **: $p < 0.01$, *: $p < 0.05$, +: $p < 0.1$.

[*]Direct correspondence to johannes.wachs@uni-corvinus.hu

VARIABLES	(1) Number of posts	(2) Number of questions	(3) Weekday posts
Stack Overflow × Post-GPT	-0.163***	-0.117*	-0.153***
	(0.0448)	(0.0460)	(0.0453)
Observations	370	370	370

Table S2: Results of a difference-in-differences model (with raw count of posts), estimating the change in activity observed weekly on Stack Overflow following the release of ChatGPT, relative to activity on four other platforms less likely to have been impacted. All regressions comprise platform fixed effects and week fixed effects. Estimation method: Poisson pseudo-maximum likelihood. The standard error of the estimate clustered on month is reported in parentheses. Significance codes: ***: $p < 0.001$, **: $p < 0.01$, *: $p < 0.05$, +: $p < 0.1$.

VARIABLES	(1) Number of posts	(2) Number of questions	(3) Weekday posts
Stack Overflow × Post-GPT	-0.178**	-0.130*	-0.164**
	(0.0478)	(0.0503)	(0.0484)
Observations	370	370	370
R2-within	0.0565	0.0454	0.0350

Table S3: Results of a difference-in-differences model (with pre-trends), estimating the change in activity observed weekly on Stack Overflow following the release of ChatGPT, relative to activity on four other platforms less likely to have been impacted. All regressions comprise platform fixed effects, week fixed effects and platform-specific pre-trends (interaction between a linear time trend and average change in the number of posts between 2018 and pre-GPT). The standard error of the estimate clustered on month is reported in parentheses. R-squared (within) is derived after differencing out week and platform fixed effects. Significance codes: ***: $p < 0.001$, **: $p < 0.01$, *: $p < 0.05$, +: $p < 0.1$.

VARIABLES	(1) Number of posts	(2) Number of questions	(3) Weekday posts
Stack Overflow × Post-GPT	-0.157**	-0.103+	-0.146**
	(0.0514)	(0.0527)	(0.0533)
Observations	1,150	1,150	1,150
R2-within	0.241	0.302	0.196

Table S4: Results of a difference-in-differences model (with pre-trends, sample from 2019), estimating the change in activity observed weekly on Stack Overflow following the release of ChatGPT, relative to activity on four other platforms less likely to have been impacted. All regressions comprise platform fixed effects, week fixed effects and platform-specific pre-trends (interaction between a linear time trend and average change in the number of posts between 2018 and pre-GPT). The standard error of the estimate clustered on month is reported in parentheses. R-squared (within) is derived after differencing out week and platform fixed effects. Significance codes: ***: $p < 0.001$, **: $p < 0.01$, *: $p < 0.05$, +: $p < 0.1$.

VARIABLES	(1)<br>5-week votes per posts<br>Upvotes	(2)<br>5-week votes per posts<br>Downvotes
SO × Post-GPT	0.0389	-0.00906
	(0.0429)	(0.00627)
Observations	292	292
R2-within	0.00131	0.00627

Table S5: Results of a difference-in-differences model, estimating the change in up- and downvoting on posts observed weekly on Stack Overflow following the release of ChatGPT, relative to voting on three other platforms (we exclude Segment Fault due to low data comparability) less likely to have been impacted. Outcome: the number of votes that posts published in a given week receive over the first five weeks, normalized to the total number of posts from this week. All regressions comprise platform fixed effects and week fixed effects. The standard error of the estimate clustered on month is reported in parentheses. R-squared (within) is derived after differencing out week and platform fixed effects. Significance codes: ***: $p < 0.001$, **: $p < 0.01$, *: $p < 0.05$, +: $p < 0.1$.

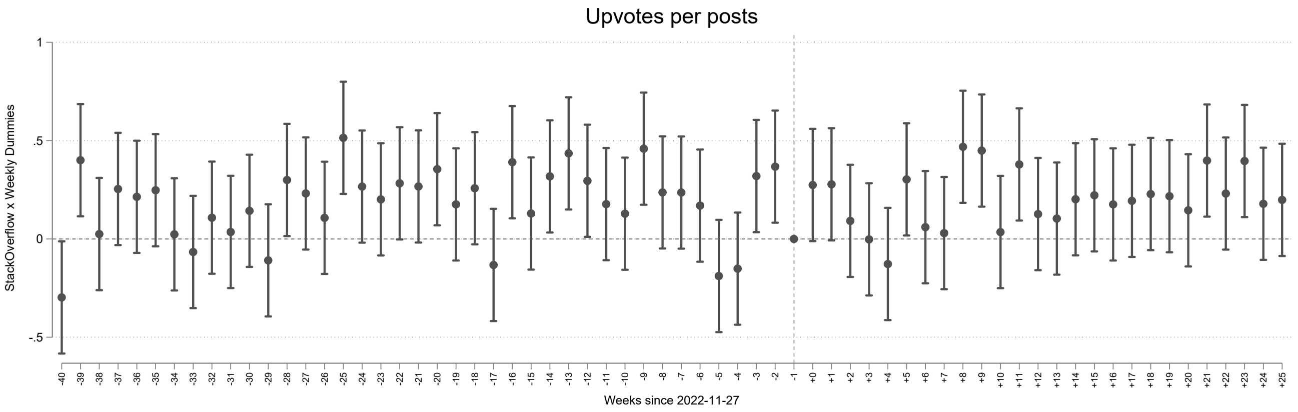

Figure S1: Difference-in-differences analysis for up-votes per post. Outcome: the number of upvotes that posts published in a given week receive over the first five weeks, normalized to the total number of posts from this week. The dashed line marks November 30, 2022 the release date of ChatGPT. The plotted coefficients correspond to the interaction between a weekly dummy and posting on Stack Overflow. The coefficients are normalized to that in the week before the release of ChatGPT. The reported confidence intervals are at 95%. The regression comprises platform fixed effects, week fixed effects, and platform-specific linear time-trends.

VARIABLES	(1)<br>Number of posts<br>Tags matched with GitHub	(2)<br>Number of posts<br>Top 50 GitHub languages
Q4 Post-GPT	-0.121**	-0.181***
	(0.0334)	(0.0399)
Q3 Post-GPT	-0.0543+	-0.114**
	(0.0269)	(0.0391)
Q2 Post-GPT	-0.0266	-0.0861*
	(0.0214)	(0.0360)
Observations	5,625	2,925
R2-within	0.00603	0.0150

Table S6: Results of a difference-in-differences model, estimating the change in posting activity observed weekly on Stack Overflow following the release of ChatGPT. We compare the change in posting between more and less popular tags (programming languages) as measured by the number of GitHub commits attributed to a given tag as of 2021. We group the 50 most popular tags into popularity quartiles based on the number of GitHub commits. $Q4$, $Q3$, and $Q2$ denote tags in the fourth, third and second popularity quartiles. Column (1) contains posting data from those tags that could be matched to GitHub. The baseline category are tags with the lowest number of pre-GPT GitHub commits. Column (2) limits the sample to tags that are among top-50 GitHub programming languages. The baseline category are tags with the lowest number of pre-GPT GitHub commits. All regressions comprise tag fixed effects and week fixed effects. The standard error of the estimate clustered on month is reported in parentheses. R-squared (within) is derived after differencing out week and platform fixed effects. Significance codes: ***: $p < 0.001$, **: $p < 0.01$, *: $p < 0.05$, +: $p < 0.1$.

VARIABLES	(1)<br>Contribute to SO ever<br>Full sample	(2)<br>Visit SO daily<br>Full sample	(3)<br>Contribute to SO weekly or more<br>Only SO contributors	(4)<br>Visit SO daily<br>Only SO contributors
Uses ChatGPT = 1	-0.056***	-0.018**	-0.041***	-0.028***
	(0.010)	(0.009)	(0.012)	(0.008)
Professional developer	0.167***	0.582***	0.080***	0.605***
	(0.012)	(0.018)	(0.016)	(0.020)
University degree	0.133***	0.134***	0.070***	0.137***
	(0.008)	(0.015)	(0.017)	(0.011)
Full-time employed	-0.152***	-0.012	-0.200***	-0.036**
	(0.011)	(0.013)	(0.014)	(0.015)
Fully remote	0.023**	-0.176***	-0.109***	-0.162***
	(0.010)	(0.008)	(0.014)	(0.007)
Years of coding	0.090***	-0.024***	-0.024***	-0.031***
	(0.003)	(0.002)	(0.003)	(0.003)
Years of coding squared	-0.001***	0.000***	0.001***	0.001***
	(0.000)	(0.000)	(0.000)	(0.000)
Observations	352,317	351,141	263,513	262,898
Clusters	49	49	49	49
Base probability (ChatGPT = 0)	0.750	0.424	0.298	0.443
Average marginal effect of ChatGPT	-0.0102	-0.00423	-0.00831	-0.00651

Table S7: Self-reported ChatGPT adoption and activity on Stack Overflow using data from the Stack Overflow Developer survey. The sample in Columns (1) and (2) includes all survey respondents who answered the questions about the frequency of their Stack Overflow visits and contributions. Columns (3) and (4), in addition, restrict the sample to SO contributors only. *Contribute to SO Ever* is a dummy variable where 1 is assigned to developers who have contributed to Stack Overflow at least once and zero otherwise. *Visit SO daily* is a dummy variable where 1 is assigned to daily users of SO, and zero otherwise. *Contribute to SO weekly or more* is a dummy variable where 1 is assigned to developers who report contributing to Stack Overflow at least several times a month. Unit of observation: respondent - language used (because most respondents report using more than one programming language). Weights are applied (1/number of languages) to avoid double counts. Estimation method: Logit (the reported coefficients represent changes in log-odds). Average marginal effect of ChatGPT (i.e. the change in the probability to visit/contribute to Stack Overflow) is shown in the last line. All specifications control for age dummies, country FE, industry FE, programming language FE and developer type. Standard errors are clustered at the programming language level. Significance codes: ***: $p < 0.001$, **: $p < 0.01$, *: $p < 0.05$, +: $p < 0.1$.

VARIABLES	(1)<br>Number of posts<br>NewUser<br>Q	(2)<br>Number of posts<br>NewUser<br>A	(3)<br>Number of posts<br>InexperiencedUser<br>Q	(4)<br>Number of posts<br>InexperiencedUser<br>A	(5)<br>Number of posts<br>ExperiencedUser<br>Q	(6)<br>Number of posts<br>ExperiencedUser<br>A	(7)<br>Number of posts<br>ExpertUser<br>Q	(8)<br>Number of posts<br>ExpertUser<br>A
Stack Overflow × Post-GPT	-0.0733+	-0.0779	-0.210**	-0.278***	-0.166**	-0.323***	-0.0647	-0.189***
	(0.0381)	(0.0584)	(0.0526)	(0.0496)	(0.0429)	(0.0462)	(0.0376)	(0.0320)
Observations	296	295	296	296	296	296	296	296
R2-within	0.0189	0.00443	0.145	0.131	0.0890	0.221	0.0107	0.0979

Table S8: Difference-in-differences models of the number of questions and answers by new, inexperienced, experienced, and expert users. The models estimate the relative impact of ChatGPT's release on posts on Stack Overflow compared to the benchmark counterfactual cases. All regressions comprise platform fixed effects and week fixed effects. Standard errors are clustered on month. R-squared (within) is derived after differencing out week and platform fixed effects. Significance codes: ***: $p < 0.001$, **: $p < 0.01$, *: $p < 0.05$, +: $p < 0.1$.
